# Dimension reduction in heterogeneous parametric spaces with application to naval engineering shape design problems

**DOI:** 10.1186/s40323-018-0118-3

**Published:** 2018-09-10

**Authors:** Marco Tezzele, Filippo Salmoiraghi, Andrea Mola, Gianluigi Rozza

**Affiliations:** Mathematics Area, mathLab, SISSA, International School of Advanced Studies, via Bonomea 265, 34136 Trieste, Italy

**Keywords:** Parametric studies, Reduction in parameter space, Free form deformation, Active subspaces, BEM, Response surface method

## Abstract

We present the results of the first application in the naval architecture field of a methodology based on active subspaces properties for parameter space reduction. The physical problem considered is the one of the simulation of the hydrodynamic flow past the hull of a ship advancing in calm water. Such problem is extremely relevant at the preliminary stages of the ship design, when several flow simulations are typically carried out by the engineers to assess the dependence of the hull total resistance on the geometrical parameters of the hull, and others related with flows and hull properties. Given the high number of geometric and physical parameters which might affect the total ship drag, the main idea of this work is to employ the active subspaces properties to identify possible lower dimensional structures in the parameter space. Thus, a fully automated procedure has been implemented to produce several small shape perturbations of an original hull CAD geometry, in order to exploit the resulting shapes and to run high fidelity flow simulations with different structural and physical parameters as well, and then collect data for the active subspaces analysis. The free form deformation procedure used to morph the hull shapes, the high fidelity solver based on potential flow theory with fully nonlinear free surface treatment, and the active subspaces analysis tool employed in this work have all been developed and integrated within SISSA *mathLab* as open source tools. The contribution will also discuss several details of the implementation of such tools, as well as the results of their application to the selected target engineering problem.

## Introduction

**Fig. 1:**
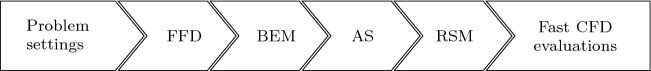
Scheme of the structure of the pipeline proposed. The geometrical deformation is performed via free from deformation (FFD), then a boundary elements method (BEM) solver computes the wave resistance, the active subspaces (AS) are detected and finally a response surface method (RSM) is employed

Nowadays engineering simulations present a wide range of different parameters. When it comes to find an optimal solution with respect to the physical constraints it is easy to be affected by the curse of dimensionality, when the number of parameters makes the simulation unfeasible. This problem arises quite easily even with a small parameter space dimension (depending on the simulation, even ten parameters could take months to be optimized). In this framework reducing the dimension of this space becomes crucial and a priority. To tackle it we focus on the active subspaces property (see [10]) to carry out a technique applied on a naval engineering problem, that is the computation of the total wave resistance of a hull advancing in calm water. In the framework of simulation-based design and shape optimization we cite, among others, [14, 16, 43, 46]. The computational pipeline we are going to present is composed first by a geometrical parametrization and deformation of the hull through free form deformation (see [40]). Then the use of a high fidelity solver based on Boundary Elements Method (BEM) to get the wave resistance with respect to the geometrical parameters. We consider also a structural parameter—the initial displacement of the hull—and a physical one—the velocity of the hull —. Subsequently active subspaces are identified thanks to the data collected from the high fidelity solver, and finally a proper reduced response surface is constructed. The result allows the final user to have an estimate of the wave resistance under a certain threshold and within a time of 1 s with respect to the hours needed for a single classic full simulation. Moreover during the process is possible to identify the most important parameters and have insights on how they influence the output of interest. Figure 1 summarizes the proposed computational pipeline.

The content of this contribution is organized as follows. “Fully nonlinear potential model” section introduces the ship resistance prediction problem, its dependence on hull shape deformations, and equations of the fluid structure interaction model used for the simulations. In “Shape morphing based on free form deformation” section we recall the free form deformation technique and we show the main features of the developed tool to manage parametric shapes. “Implementation of high fidelity potential solver based on the boundary element method” section has the purpose of introducing some detail about the high fidelity solver implementation. In “Parameter space reduction by active subspaces” section we present the active subspaces properties and its features, with a numerical recipe to identify them. Then “Numerical results” section shows the numerical results obtained by coupling the three methods in sequence. Finally conclusions and perspectives are drawn in “Conclusions and perspectives” section.

## A model naval problem: wave resistance estimation of a hull advancing in calm water

**Fig. 2:**
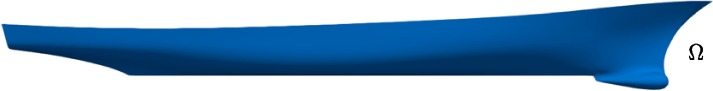
Representation of the reference domain $\varOmega$, that is the DTMB 5415 hull

In this section we introduce the problem of the estimation of the resistance of a hull advancing in calm water. The model hull shape considered in this work is the DTMB 5415, which was originally conceived for the preliminary design of a US Navy Combatant ship. Due to the wealth of experimental data available in the literature (see for example [33, 42]) such shape, which includes a sonar dome and a transom stern (see Fig. 2), has become a common benchmark for naval hydrodynamics simulation tools.

Let $\varOmega \subset {\mathbb {R}}^3$, be a domain (see Fig. 2) associated with our DTMB 5415 model hull. We call $\varOmega$ the *reference* domain; for practical reasons this domain happens to correspond to the undeformed hull, even though this assumption is not fundamental for the remainder of the paper. We here remark that the domain considered in the fluid dynamic simulations is in principle the volume surrounding the hull which is occupied by water, namely $\varOmega _w$. Further details about the fluid dynamic domain will be provided in “Fully nonlinear potential model” section.

Let ${\mathcal {M}}(\varvec{x}; \varvec{\mu }^{\text {GEOM}}): {\mathbb {R}}^3 \rightarrow {\mathbb {R}}^3$ be a shape morphing that maps the reference domain $\varOmega$ into the deformed domain $\varOmega (\varvec{\mu }^{\text {GEOM}})$ as follows:

$$\begin{aligned} \varOmega (\varvec{\mu }^{\text {GEOM}}) = {\mathcal {M}}(\varOmega ; \varvec{\mu }^{\text {GEOM}}). \end{aligned}$$

Quite naturally, the results of the fluid dynamic simulations will depend on the specific hull shape considered, which are in turn associated to the parameters defining the morphing ${\mathcal {M}}$ (which will be extensively defined in “Shape morphing based on free form deformation” section). It is worth pointing out here that the geometrical quantity having the most effect on the resistance is the immersed volume of a hull shape, as higher volumes will generate higher drag values. This is clearly due to the fact that higher hull volumes will result in a higher mass of water displaced as the ship advances in the water, and in increased surface exposed to the water friction. This consideration might lead to the naive conclusion that since the shape optimizing the total drag is the one corresponding to zero buoyant volume, the hull volume must be constrained in the optimization algorithm, to avoid a convergence to such trivial shape, which would not generate a vertical force able to sustain the ship weight. In this work, rather than constraining the hull volume through more complex hull deformation algorithms, we decided to impose the weight (or displacement) of the hull in the fluid dynamic simulations, so that each hull would reach its hydrostatic equilibrium position, in which the weight prescribed at the design stage is balanced by the vertical hydrodynamic force. This solution, which of course requires a model accounting for the rigid motions of the hull into the fluid dynamic simulations, is able to lead to design solutions which optimize the total resistance while retaining the required load capability of the ship.

For all the aforementioned reasons, along with the geometrical parameters associated with the hull morphing, the results of our simulations are also affected by the ship displacement and cruise speed, which are instead physical parameters determining the hydrodynamic equilibrium position and forces. Thus, considering both the geometric morphing and variations in the physical parameters, we have a set of $m\in {\mathbb {N}}$ parameters which affect the output of the fluid dynamic simulations. The parametric domain considered is then defined as ${\mathbb {D}} \subset {\mathbb {R}}^m$, and is assumed to be a box in ${\mathbb {R}}^m$.

By a practical standpoint, once a point in the parameter domain ${\mathbb {D}}$ is identified, the specific hull geometry as well as the desired ship displacement and cruise speed are provided to the fluid dynamic solver, which carries out a flow simulation to provide a resistance estimate. In this framework free form deformation has been employed for the generation of a very large number of hull geometries obtained from the morphing of the DTMB 5415 naval combatant hull. Each geometry generated has been used to set up a high fidelity hydrodynamic simulation with the desired ship displacement and hull speed. The output resistances for all the configurations tested have been finally analyzed by means of active subspaces in order to reduce the parameter space.

In the next subsections, we will provide a brief description of the unsteady fully nonlinear potential fluid dynamic model used to carry out the high fidelity simulations. In addition, we will describe the rigid body equations based on hull quaternions used to compute the hull linear and angular displacements corresponding to the final hydrodynamic equilibrium position reached at the end of each simulation. We refer the interested reader to [29–31] for further information on the fully nonlinear potential free surface model, on its application to complex hull geometries, and on the treatment of the hull rigid motions, respectively.

### Fully nonlinear potential model

In the simulations we are only considering the motion of a ship advancing at constant speed in calm water. For such reason we solve the problem in a *global*, translating reference frame ${\widehat{XYZ}}$, which is moving with the constant horizontal velocity of the boat $\varvec{V}_\infty = (V_\infty ,0,0)$. Thus, the *X* axis of the reference frame is aligned with $\varvec{V}_\infty$, the *Z* axis is directed vertically (positive upwards), while the *Y* axis is directed laterally (positive port side).

As aforementioned, the domain $\varOmega _w(t)$ in which we are interested in computing the fluid velocity and pressure is represented by the portion of water surrounding the ship hull. The time varying shape of such domain—and in particular that of its boundary with the air above—is one of the unknowns of the fluid dynamic problem. By convention, we place the origin of the vertical axis *Z* in correspondence with the undisturbed free surface level, and we start each simulation at time $t=0$ from such undisturbed configuration. Thus, at least in its initial configuration, the flow domain is represented by $\varOmega _w(t=0) = {\mathbb {R}}^3_{Z-}\backslash \varOmega$, which is the boolean subtraction of the hull volume from the lower half-space of ${\mathbb {R}}^3$ for which $Z\le 0$, here indicated with ${\mathbb {R}}^3_{Z-}$.

If overturning waves are not observed (which is typically the case for low cruise velocities typical of a ship), the domain $\varOmega _w(t)$ is simply connected. So under the assumptions of irrotational flow and non viscous fluid the velocity field $\varvec{v}(\varvec{X},t)$ admits a representation through a scalar potential function $\varPhi (\varvec{X},t)$, namely

1 $$\begin{aligned} \varvec{v}= {\varvec{\nabla }}\varPhi = {\varvec{\nabla }}(\varvec{V}_\infty \cdot \varvec{X}+ \phi ) \qquad \qquad \forall \ \varvec{X}\in \varOmega _w(t), \end{aligned}$$

in which $\phi (\varvec{X},t)$ is the *perturbation potential*. Under the present assumptions, the equations of motion simplify to the unsteady Bernoulli equation and to the Laplace equation for the perturbation potential:

2a $$\begin{aligned}&\frac{\partial \phi }{\partial t}+\frac{1}{2}\left| {\varvec{\nabla }}\phi +\varvec{V}_\infty \right| ^2 +\frac{p-p_a}{\rho }-\varvec{g}\cdot \varvec{X}= C(t) \qquad \text { in } \varOmega _w(t) , \end{aligned}$$

2b $$\begin{aligned}&\varDelta \phi = 0 \qquad \text { in } \varOmega _w(t), \end{aligned}$$

where *C*(*t*) is an arbitrary function of time, and $\varvec{g}= (0,0,-g)$, is the gravity acceleration vector, directed along the *z* axis. The unknowns of such mathematical problem $\phi$ and *p* are uncoupled. This means that the solution of the Poisson problem in Eq. (2b) can be obtained independently of the pressure field. Once such solution is obtained, the pressure can be obtained through a postprocessing step based on Bernoulli Eq. (2a). Thus, the Laplace equation is the governing equation of our model. Such equation is complemented by non penetration boundary conditions on the hull surface $\varGamma ^{b}(t)$ and water basin bottom boundary $\varGamma ^{bot}(t)$, and by homogeneous Neumann boundary conditions on the truncation boundaries $\varGamma ^{far}(t)$ of the numerical domain. The bottom of the basin is located at a depth corresponding to 2 boat lengths, while the truncation boundaries are located approximatively at a distance from the boat of 6 boat lengths in the longitudinal direction *X* and of 2 boat lengths in the lateral direction *Y*). On the water free surface $\varGamma ^{w}(t)$, we employ the kinematic and dynamic Semi-Lagrangian fully nonlinear boundary conditions, which respectively read

3 $$\begin{aligned} \frac{\delta \eta }{\delta t}= & {} \frac{\partial \phi }{\partial z}+{\varvec{\nabla }}\eta \cdot \left( \varvec{w}-{\varvec{\nabla }}\phi -\varvec{V}_\infty \right) \qquad \text { in } \varGamma ^{w}(t) , \end{aligned}$$

4 $$\begin{aligned} \frac{\delta \phi }{\delta t}= & {} -g\eta + \frac{1}{2}|{\varvec{\nabla }}\phi |^2 + {\varvec{\nabla }}\phi \cdot \left( \varvec{w}-{\varvec{\nabla }}\phi -\varvec{V}_\infty \right) \qquad \text { in } \varGamma ^{w}(t). \end{aligned}$$

The former equation expresses the fact that a material point moving on the free surface will stay on the free surface—here assumed to be a single valued function $\eta (X,Y,t)$ of the horizontal coordinates *X* and *Y*. The latter condition is derived from Bernoulli Eq. (2a), under the assumption of constant atmospheric pressure on the water surface. This peculiar form of the fully nonlinear boundary conditions was proposed by Beck [4]. Eq. (3) allows for the computation of the vertical velocity of markers which move on the water free surface with a prescribed horizontal speed $(w_X,w_Y)$. Eq. (4) is used to obtain the velocity potential values in correspondence with such markers. The resulting vector $\varvec{w}=(w_X,w_Y,\frac{\delta \eta }{\delta t}) = {\dot{\varvec{X}}}$ is the time derivative of the position of the free surface markers. In this work, such free surface markers are chosen as the free surface nodes of the computational grid. To avoid an undesirable mesh nodes drift along the water stream, the markers arbitrary horizontal velocity is set to 0 along the *X* direction. The *Y* component of the water nodes in contact with the ship—which is moved according with the computed linear and angular displacements—is chosen so as to keep such nodes on the hull surface. As for the remaining water nodes, the lateral velocity value is set to preserve mesh quality.

### Three dimensional hull rigid motions

**Fig. 3:**
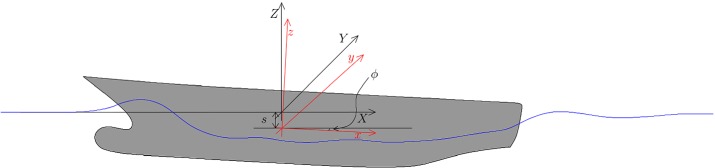
A sketch illutrating the hull-attached frame ${\widehat{xyz}}$ in red and the global reference frame ${\widehat{XYZ}}$ which is moving with the constant horizontal velocity of the boat. The ship here depicted is experiencing a vertical displacement *s* and an angular displacement characterized by the pitch angle $\phi$

The ship hull is assumed to be a rigid body. A second, *hull-attached* reference frame ${\widehat{xyz}}$, which follows the hull in its translations and rotations is employed to study the ship motions. The center of such reference frame is located at the ship center of gravity, which in the global reference frame reads $\varvec{X}^G(t) = X^G(t)\varvec{e}_X+Y^G(t)\varvec{e}_Y+Z^G(t)\varvec{e}_Z$, where $\varvec{e}_X$, $\varvec{e}_Y$, $\varvec{e}_Z$ are the unit vectors along the global system axes. See Fig. 3 for a detailed sketch.

The rotation matrix *R*(*t*) is used to convert the coordinates of a point $\varvec{x}$ written in the hull-attached reference frame, to those in the global frame $\varvec{X}$, namely

5 $$\begin{aligned} \varvec{X}(t) = R(t)\varvec{x}+\varvec{X}^G(t). \end{aligned}$$

The global frame velocity of a point having coordinates $\varvec{x}$ in the hull-attached frame is obtained as

6 $$\begin{aligned} \varvec{V}^{\text {hp}}(t) = {\varvec{\omega }}(t)\times \varvec{x}+{\dot{\varvec{X}}}^G(t), \end{aligned}$$

in which ${\varvec{\omega }}$ is the angular velocity vector.

Equations (5) and (6) imply that once $\varvec{X}^G(t)$, *R*(*t*), and ${\varvec{\omega }}(t)$ are known at time *t*, the position and velocity of each point of the hull can be obtained. For this reason, writing the time evolution equations for $\varvec{X}^G(t)$, *R*(*t*), and ${\varvec{\omega }}(t)$ is sufficient to fully determine the hull dynamics. In the next sections, we will present such evolution equations written in the global reference frame, as presented in [1, 17].

The evolution equation for $\varvec{X}^G(t)$ is obtained via the linear momentum conservation equation, which in the case of our hydrodynamics simulation framework reads

7 $$\begin{aligned} m_s\ddot{\varvec{X}}^G(t) = m_s\varvec{g}+ \varvec{F}^w(t). \end{aligned}$$

In Eq. (7), $m_s$ is the mass of the ship, while the hydrodynamic force vector $\varvec{F}^w(t)$ is in the present work obtained as the sum of the pressure and viscous forces on the hull.

The evolution equation for ${\varvec{\omega }}$ is obtained writing the angular momentum conservation, namely

8 $$\begin{aligned} R(t) I^G R(t)^T{\dot{{\varvec{\omega }}}}(t) + {\varvec{\omega }}(t) \times R(t) I^G R(t)^T {\varvec{\omega }}(t) = \varvec{M}^w(t), \end{aligned}$$

where $I^G$ is the matrix of inertia of the ship in the hull-attached reference frame, and hydrodynamic moment vector $\varvec{M}^w(t)$ is the sum of the moment about the ship center of gravity of the pressure and viscous forces on hull, propeller and appendages.

To write an evolution equation for *R*, starting from the angular velocity vector ${\varvec{\omega }}(t)=[\omega _X(t),\omega _Y(t),\omega _Z(t)]$, we first introduce the skew symmetric tensor

9 $$\begin{aligned} \omega (t) = \left[ \begin{array}{c c c} 0 &{} -\omega _Z(t) &{} \omega _Y(t) \\ \omega _Z(t) &{} 0 &{} -\omega _X(t) \\ -\omega _Y(t) &{} \omega _X(t) &{} 0 \end{array}\right] . \end{aligned}$$

Note that tensor $\omega (t)$ will act on a vector $\varvec{u}\in {\mathbb {R}}^3$ as if the vector product by ${\varvec{\omega }}(t)$ were applied to $\varvec{u}$:

10 $$\begin{aligned} {\varvec{\omega }}(t)\times \varvec{u}= \omega (t)\varvec{u}. \end{aligned}$$

Making use of such tensor, an evolution equation for the rotation matrix *R*(*t*) reads

11 $$\begin{aligned} {\dot{R}}(t) = \omega (t) R(t), \end{aligned}$$

which can be advanced in time to obtain the components of *R* and close the equations of motions of a rigid body in three dimension. Yet, in the common practice of rigid body simulations, direct numerical integration of Eq. (11) is avoided. The most important reason for this is related to numerical drift. If we in fact keep track of the orientation of a rigid body integrating Eq. (11), numerical error will build up in the entries of *R*(*t*), so that it will no longer be a rotation matrix, losing its properties of orthogonality and of having determinant equal to 1. Physically, the effect would be that applying *R*(*t*) to a body would cause a skewing effect.

A better way to represent the orientation of a rigid body in three dimensions (even with large rotations) is represented by the use of *unit quaternions* (see the work of [41] for details). For our purposes, quaternions can be considered as a particular type of four element vector, normalized to unit length. If we indicate the quaternion $\varvec{q}= s + v_X\varvec{e}_X + v_Y\varvec{e}_Y + v_Z\varvec{e}_Z$ as $\left[ s,\varvec{v}\right]$, the internal product of two quaternions $\varvec{q}_1$ and $\varvec{q}_2$ is defined as

12 $$\begin{aligned} \varvec{q}_1\varvec{q}_2 = \left[ s_1,\varvec{v}_1\right] \left[ s_2,\varvec{v}_2\right] = \left[ s_1s_2-\varvec{v}_1\cdot \varvec{v}_2\, , \, s_1\varvec{v}2+s_2\varvec{v}1+\varvec{v}_1\times \varvec{v}_2\right] . \end{aligned}$$

The norm of a quaternion $\varvec{q}$ is defined as $||\varvec{q}|| = \sqrt{s^2+v_X^2+v_Y^2+v_Z^2}$. Unit quaternions can be used to represent rotations in a three dimensional space. In fact, given a quaternion $\varvec{q}(t): ||\varvec{q}(t)||=1 \quad \forall t$, we can obtain the corresponding rotation matrix as

13 $$\begin{aligned} R = \left[ \begin{array}{c c c} 1-2v_Y^2-2v_Z^2 &{} 2v_Xv_Y-2s v_Z &{} 2v_Xv_Z+2s v_Y \\ 2v_Xv_Y+2s v_Z &{} 1-2v_Y^2-2v_Z^2 &{} 2v_Yv_Z-2s v_X \\ 2v_Xv_Z-2s v_Y &{} 2v_Yv_Z+2s v_X &{} 1-2v_Y^2-2v_Z^2 \end{array}\right] , \end{aligned}$$

in which to lighten the notation we omitted the time dependence of both *R*(*t*) and the components of $\varvec{q}(t)$.

Finally, the equation needed to describe the time evolution for the hull quaternion $\varvec{q}(t)$ is

14 $$\begin{aligned} {\dot{\varvec{q}}}(t) = \frac{1}{2}{\varvec{\omega }}_q(t)\varvec{q}(t), \end{aligned}$$

where ${\varvec{\omega }}_q(t) = \left[ 0,{\varvec{\omega }}(t)\right]$ is the quaternion associated with the angular velocity vector ${\varvec{\omega }}(t)$. As quaternions only have four entries, there only is one extra variable used to describe the three degrees freedoms of a three dimensional rotation. A rotation matrix instead employs nine parameters for the same three degrees of freedom; thus, the quaternions present far less redundancy than rotation matrices. Consequently, quaternions experience far less numerical drift than rotation matrices. The only possible source of drift in a quaternion occurs when the quaternion has lost its unit magnitude. This can be easily corrected by periodically renormalizing the quaternion to unit length [41].

## Shape morphing based on free form deformation

As already mentioned, we are interested in problems characterized by both physical and geometrical parameters. In such framework, the free form deformation (FFD) approach is adopted to implement the hull deformations corresponding to each geometrical parameter set considered.

A very detailed description of FFD is beyond the scope of the present work. In the following we will give only a brief overview. For a further insight see [40] for the original formulation and [18, 26, 36, 38, 39] for more recent works.

We decided to adopt free form deformation among other possibilities (including, for instance, radial basis functions or inverse distance weighting) because it allows to have global deformations with a few parameters. For the complexity of the problem at hand, by trying to reduce the number of parameters starting from hundreds of them can be infeasible for the number of Monte Carlo simulations required. One of the possible drawbacks of FFD is generally that the parameters do not have a specific geometric meaning, like, for instance, a prescribed length or distance. In the case of application to active subspaces (AS) this is not a problem since AS itself identifies new parameters, obtained by combination of the original ones, meaningless from the geometric and physical point of view.

**Fig. 4:**
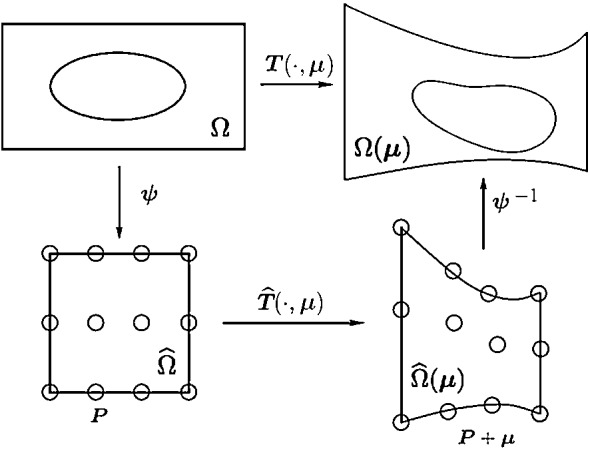
Sketch of the FFD map construction. For ease of readability we dropped the superscript from $\varvec{\mu }^{\text {GEOM}}$

Free from deformation consists basically in three different step, as shown in Fig. 4:

- Mapping the physical domain $\varOmega$ to the reference one ${\widehat{\varOmega }}$ with the map $\varvec{\psi }$.
- Moving some control points $\varvec{P}$ to deform the lattice with ${\widehat{T}}$. The movement of the control points is given by the weights of FFD, and represent our geometrical parameters $\varvec{\mu }^{\text {GEOM}}$.
- Mapping back to the physical domain $\varOmega (\varvec{\mu })$ with the map $\varvec{\psi }^{-1}$.

So FFD map *T* is the composition of the three maps, i.e.

15 $$\begin{aligned} T(\cdot , \varvec{\mu }^{\text {GEOM}}) = (\varvec{\psi }^{-1} \circ {\widehat{T}} \circ \varvec{\psi }) (\cdot , \varvec{\mu }^{\text {GEOM}}) . \end{aligned}$$

In particular, in the three dimensional case, for every point $\varvec{X} \in \varOmega$ inside the FFD box, its position changes according to

16 $$\begin{aligned} T(\varvec{X}, \varvec{\mu }^{\text {GEOM}}) = \varvec{\psi }^{-1} \left( \sum _{l=0} ^L \sum _{m=0} ^M \sum _{n=0} ^N b_{lmn}(\varvec{\psi }(\varvec{X})) \varvec{P}_{lmn}^0 \left( \varvec{\mu }^{\text {GEOM}}_{lmn}\right) \right) , \end{aligned}$$

where $b_{lmn}$ are Bernstein polynomials of degree *l*, *m*, *n* in each direction, respectively, $\varvec{P}_{lmn}^0 \left( \varvec{\mu }^{\text {GEOM}}_{lmn}\right) = \varvec{P}_{lmn} + \varvec{\mu }^{\text {GEOM}}_{lmn}$, and $\varvec{P}_{lmn}$ represents the coordinates of the control point identified by the three indices *l*, *m*, *n* in the lattice of control points. We also explicit the ${\hat{T}}$ mapping as follows

$$\begin{aligned} {\hat{T}}(\varvec{Y}, \varvec{\mu }^{\text {GEOM}}) := \sum _{l=0} ^L \sum _{m=0} ^M \sum _{n=0} ^N b_{lmn}(\varvec{Y}) \varvec{P}_{lmn}^0 \left( \varvec{\mu }^{\text {GEOM}}_{lmn}\right) \qquad \forall \, \varvec{Y} \in {\widehat{\varOmega }}. \end{aligned}$$

**Fig. 5:**
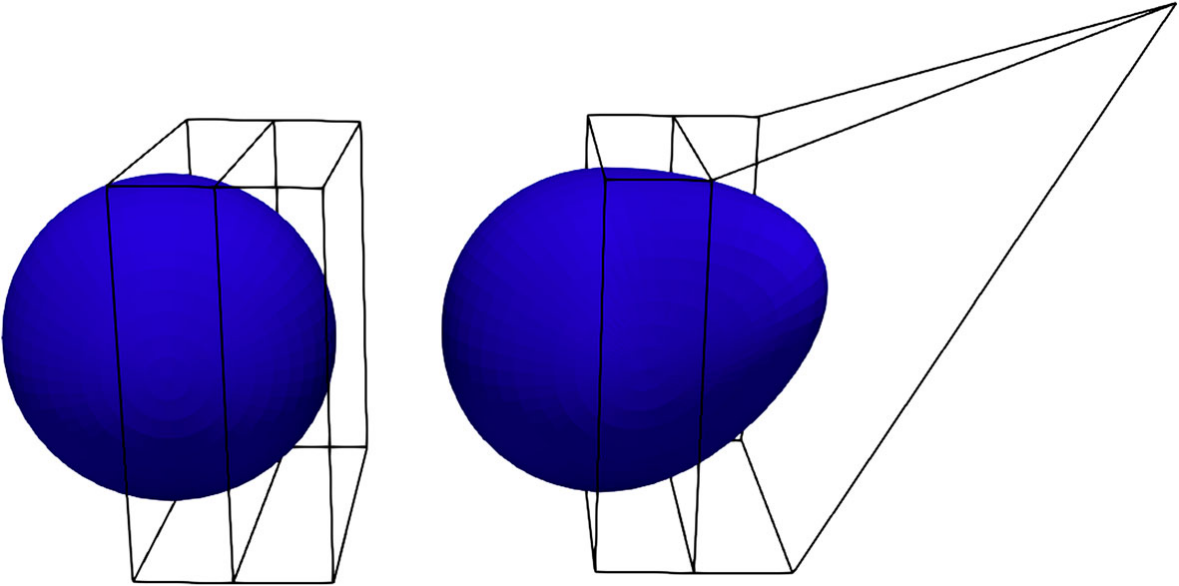
FFD morphing on a simple geometry: the sphere case. Here we move only one FFD control point in the lattice

In Fig. 5, we show, for example, the application of the FFD morphing on a very simple geometry, that is a sphere.

We implemented this algorithm in a *python* package called *PyGeM* [35] in order to deal with the major industrial CAD file formats. It handles *iges*, *stl*, *step*, *vtk*, *unv*, *keyword*, and *openfoam* files. It extracts the coordinates of the control points, deforms them according to the inputs given by the user and writes a new file with the morphed CAD. We improve the traditional version of the algorithm by allowing a rotation of the FFD lattice in order to give more flexibility to the tool. In general with our package it is possible to have a generic bounding box (not only a cube) as long as the $\varvec{\psi }$ map is affine.

**Fig. 6:**
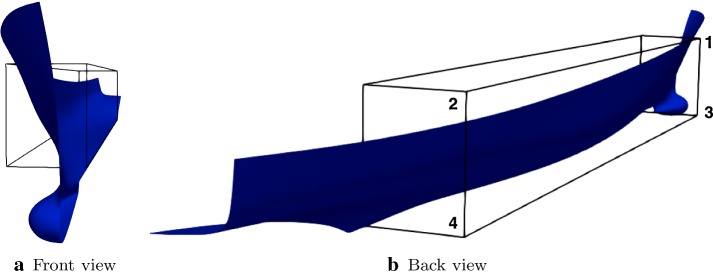
Plot **a** shows the FFD lattice over one side wall of the hull from the front, while plot **b** depicts the hull and the lattice from the back together with the numbers that identify the FFD points

In order to exemplify the equations above to our case, let us consider Fig. 6, where the control points we are going to move are marked with numbers. As geometrical parameters we select six components of these four control points of the FFD lattice over one side wall of the hull. Then we apply the same deformation to the other side. This because one of our hypothesis is the symmetry of the deformed hull. In particular Table 1 summarizes the set of design variables, the associated FFD-node coordinate modified (*y* is the span of the hull, *x* its length and *z* its depth) and the lower and upper bound of the modification. There are also two more parameters that do not affect the geometry, and are related to the physics of the problem, that is the displacement and the velocity of the hull. From now on we denote with $\varvec{\mu } := \{ \mu _i \}_{i \in [1, \dots , 8]}$ the column vector of the parameters, where $\mu _i$ are defined in Table 1. To denote only the parameters affecting the geometrical deformation we use $\varvec{\mu }^{\text {GEOM}} := \{ \mu _i \}_{i \in [1, \dots , 6]}$. For sake of clarity we underline that the undeformed original domain is obtained setting all the geometrical parameters to 0. All the upper and lower bounds are chosen in order to satisfy physical constraints.

To create the dataset with all the deformation, we started from the original *iges* file of the hull and deformed it with the *PyGeM* package. The deformations are generated randomly with an uniform distribution.

**Table 1:** Design space (FFD lattice $2 \times 2 \times 2$) with eight design parameters

**Parameter**	**Nature**	**Lower bound**	**Upper bound**
$\mu _1$	FFD point 1 *y*	$-$ 0.2	0.3
$\mu _2$	FFD point 2 *y*	$-$ 0.2	0.3
$\mu _3$	FFD point 3 *y*	$-$ 0.2	0.3
$\mu _4$	FFD point 4 *y*	$-$ 0.2	0.3
$\mu _5$	FFD point 3 *z*	$-$ 0.2	0.5
$\mu _6$	FFD point 4 *z*	$-$ 0.2	0.5
$\mu _7$	Weight (kg)	500	800
$\mu _8$	Velocity (m/s)	1.87	2.70

Six geometrical parameters chosen among the FFD control points, one structural parameter that is the initial displacement of the hull and one physical parameter given by the velocity

## Implementation of high fidelity potential solver based on the boundary element method

The boundary value problem described in “Fully nonlinear potential model” section is governed by the linear Laplace operator. Yet, it is nonlinear due to the presence of the boundary conditions in Eqs. (3) and (4). Further sources of nonlinearity are given by continuous change of the domain shape over time and by the arbitrary shape of the ship hull. Thus, for each time instant, we will look for the correct values of the unknown potential and node displacement fields by solving a specific nonlinear problem resulting from the spatial and time discretization of the original boundary value problem. The spatial discretization of the Laplace problem is based upon a boundary integral formulation described in [19]. In this framework, the domain boundary is subdivided into quadrilateral cells, on which bi-linear shape functions are used to approximate the surface, the flow potential values, and the normal component of its surface gradient. The resulting Boundary Element Method (BEM, see [6]) consists in collocating a boundary integral equation (BIE) in correspondence with each node of the numerical grid, and computing the integrals appearing in such equation by means of the described iso-parametric formulation. The linear algebraic equations obtained from such discretization method are combined with the Ordinary Differential Equations (ODE) derived from the Finite Element spatial discretization of the fully nonlinear free surface boundary conditions in Eqs. (3) and (4). The spatial discretization described is carried out making use of the deal.II open source library for C++ implementation of finite element discretizations [2, 3]. To enforce a strong coupling between the fluid and structural problem, the aforementioned system of Differential Algebraic Equations (DAE) is complemented by the equations of the rigid hull dynamics [(Eqs. (7), (8) and (14)]. The DAE system solution is time integrated by means of an arbitrary order and arbitrary time step implicit backward difference formula (BDF) scheme implemented in the IDA package of the open source C++ library SUNDIALS [21]. The potential flow model described has been implemented in a stand alone C++ software, the main features of which are described in [30].

**Fig. 7:**
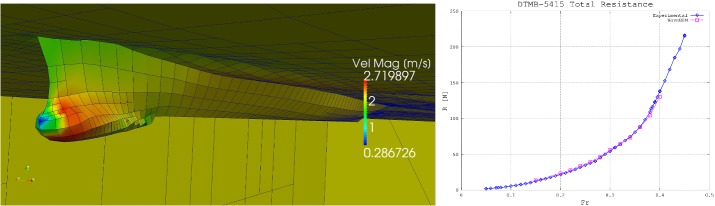
On the left, the mesh automatically generated on the surface of one of the deformed hulls obtained starting from the DTMB 5415 Navy Combatant Hull. On the right, the total resistance of the DTMB 5415 hull as a function of the Froude number associated with the surge velocity imposed in the simulations. The blue continuous line represents the experimental values presented in Olivieri et al. [33]. The values obtained in this work are represented by the dashed magenta line

The solver is complemented with a mesh module directly interfaced with CAD data structures [31]. Such feature allows for fully automated mesh generation once a hull shape is assigned at the start of each simulation by means of a—possibly non water-tight—CAD geometry. Figure 7, on the left, displays the mesh generated on the surface of a DTMB 5415 Navy Combatant hull. At each time step of the simulation, the wave resistance is computed as

17 $$\begin{aligned} R^w = \int _{\varGamma ^b}p\varvec{n}\,d\varGamma \cdot \varvec{e}_X, \end{aligned}$$

where *p* is the pressure value obtained introducing the computed potential in Eq. (2a). The inviscid fluid dynamic model drag prediction is then corrected by adding a viscous drag contribution obtained by means of the ITTC-57 formula [32]. A full assesment of the accuracy of the high fidelity fluid structure interaction sover described is clearly beyond the scope of the present work (again, we refer the interested reader to [29–31] for more details). Yet, in Fig. 7, on the right, we present a comparison between the computed total resistance curve and the corresponding one measured by Olivieri et al. [33]. As it can be appreciated in the plot, for all the Froude numbers tested the computed total drag difference with respect to the measurements is less then 6%. Given the fact that all the geometries tested are deformations of the present hull, and that all the velocities imposed fall in the range reported in the plot, it is reasonable to infer that for each simulation carried out the accuracy of the high fidelity model prediction will be similar to that of the results presented.

## Parameter space reduction by active subspaces

The active subspaces (AS) approach represents one of the emerging ideas for dimension reduction in the parameter studies and it is based on the homonymous properties. The concept was introduced by Constantine in [10], for example, and employed in different real world problems. We mention, among others, aerodynamic shape optimization [27], the parameter reduction for the HyShot II scramjet model [12], active subspaces for integrated hydrologic model [23], and in combination with POD-Galerkin method in cardiovascular problems [44].

**Fig. 8:**
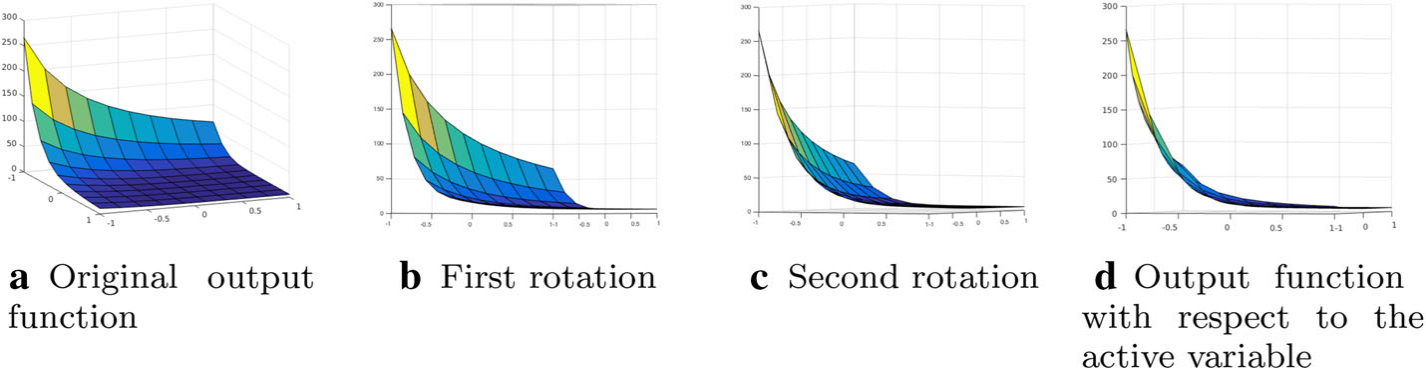
Example of a bivariate output function (**a**), intermediate rotations of the domain (**b**) and (**c**), and the final state (**d**), where we can see the variation of the function along the active variable

A characteristic of the active subspaces is that they identify a set of important directions in the space of all inputs, instead of identifying a subset of the inputs as important. If the simulation output does not change as the inputs move along a particular direction, then we can safely ignore that direction in the parameter study. In Fig. 8 it is possible to capture the main idea behind the active subspaces approach: we try to rotate the inputs domain in such a way lower dimension behavior of the output function is revealed. When an active subspace is identified for the problem of interest, then it is possible to perform different parameter studies such as response surfaces [5], integration, optimization and statistical inversion [24].

There are some main ingredients in order to employ active subspaces. The first is a scalar function $f: {\mathbb {R}}^m \rightarrow {\mathbb {R}}$ smooth enough depending on the inputs $\varvec{\mu }$ that represents the quantity of interest. Moreover we need the gradients of this map with respect to the inputs $\nabla _{\varvec{\mu }} f(\varvec{\mu })$ or an approximation of them, a sampling density $\rho$, and a gap between eigenvalues of the covariance matrix associated to the gradients. That is a symmetric, positive semidefinite matrix whose elements are the average products of partial derivatives of the simulations’ input/output map, that reads

18 $$\begin{aligned} \varvec{\varSigma } = {\mathbb {E}}\, [\nabla _{\varvec{\mu }} f \, \nabla _{\varvec{\mu }} f ^T] = \int _{{\mathbb {D}}} (\nabla _{\varvec{\mu }} f) ( \nabla _{\varvec{\mu }} f )^T \rho \, d \varvec{\mu } , \end{aligned}$$

where ${\mathbb {E}}$ is the expected value, $\rho : {\mathbb {R}}^m \rightarrow {\mathbb {R}}^+$ a probability density function — usually a uniform one —, and $\varvec{\varSigma }$ the so-called uncentered covariance matrix of the gradients of *f* (for a more deep understanding of these operators see for example [15]). Usually a Monte Carlo method (see [28]) is used in order to approximate the eigenpairs of this matrix (see [8]) as follows

19 $$\begin{aligned} \varvec{\varSigma } \approx \frac{1}{N_{\text {train}}^{\text {AS}}} \sum _{i=1}^{N_{\text {train}}^{\text {AS}}} \nabla _{\varvec{\mu }} f_i \, \nabla _{\varvec{\mu }} f^T_i , \end{aligned}$$

where we draw $N_{\text {train}}^{\text {AS}}$ samples $\varvec{\mu }^{(i)}$ independently from the measure $\rho$ and where $\nabla _{\varvec{\mu }} f_i = \nabla _{\varvec{\mu }} f(\varvec{\mu }^{(i)})$. This matrix is symmetric and positive semidefinite, so it admits a real eigenvalue decomposition

20 $$\begin{aligned} \varvec{\varSigma } = {\mathbf {W}} \varvec{\Lambda } {\mathbf {W}}^T , \end{aligned}$$

where ${\mathbf {W}}$ is a $m \times m$ column matrix of eigenvectors, and $\varvec{\Lambda }$ is a diagonal matrix of eigenvalues. Then we order the eigenpairs in descending order. We will select the first *M* eigenvectors to form a reduced-order basis. This is where we reduce the dimensionality of the design problem. We will attempt to describe the behaviour of the objective function by projecting the full-space design variables onto this active subspace. On average, perturbations in the first set of coordinates change *f* more than perturbations in the second set of coordinates. Low eigenvalues suggest that the corresponding vector is in the nullspace of the covariance matrix, and to form an approximation we can discard these vectors. We select the basis as follow

21 $$\begin{aligned} \varvec{\Lambda } = \begin{bmatrix} \varvec{\Lambda }_1&\\&\varvec{\Lambda }_2\end{bmatrix}, \qquad {\mathbf {W}} = \left[ {\mathbf {W}}_1 \quad {\mathbf {W}}_2 \right] , \end{aligned}$$

where $\varvec{\Lambda }_1 = diag(\lambda _1, \dots , \lambda _M)$ with $M<m$, and ${\mathbf {W}}_1$ contains the first *M* eigenvectors. The active subspace is the span of the first few eigenvectors of the covariance matrix. We define the active variables to be the linear combinations of the input parameters with weights from the important eigenvectors. In particular we define the active subspace to be the range of ${\mathbf {W}}_1$. The inactive subspace is the range of the remaining eigenvectors in ${\mathbf {W}}_2$. With the basis identified, we can map forward to the active subspace. So $\varvec{\mu }_M$ is the active variable and $\varvec{\nu }$ the inactive one, respectively:

22 $$\begin{aligned} \varvec{\mu }_M = {\mathbf {W}}_1^T\varvec{\mu } \in {\mathbb {R}}^M, \qquad \varvec{\nu } = {\mathbf {W}}_2^T \varvec{\mu } \in {\mathbb {R}}^{m-M} . \end{aligned}$$

In particular any point in the parameter space $\varvec{\mu } \in {\mathbb {R}}^m$ can be expressed in terms of $\varvec{\mu }_M$ and $\varvec{\nu }$:

$$\begin{aligned} \varvec{\mu } = {\mathbf {W}}{\mathbf {W}}^T\varvec{\mu } = {\mathbf {W}}_1{\mathbf {W}}_1^T\varvec{\mu } + {\mathbf {W}}_2{\mathbf {W}}_2^T\varvec{\mu } = {\mathbf {W}}_1 \varvec{\mu }_M + {\mathbf {W}}_2 \varvec{\nu } . \end{aligned}$$

Having this decomposition in mind we can rewrite *f*

$$\begin{aligned} f (\varvec{\mu }) = f ({\mathbf {W}}_1 \varvec{\mu }_M + {\mathbf {W}}_2 \varvec{\nu }) , \end{aligned}$$

and construct a surrogate model *g* discarding the inactive variables

$$\begin{aligned} f (\varvec{\mu }) \approx g ({\mathbf {W}}_1^T \varvec{\mu }) = g(\varvec{\mu }_M). \end{aligned}$$

The surrogate model *g* in this work is a response surface. We exploit the decreased number of parameters to fit a lower dimensional response surface, fighting the curse of dimensionality that affects this approximation procedure. The advantage of this approach is that more models are feasible, such as for example radial basis functions interpolation, higher degree polynomials, or regressions techniques. In particular we use a polynomial response surface. For different type of surrogate model that exploit a shared active subspaces in naval engineering refer to [45].

We underline that the size of the eigenvalue problem is the limiting factor. We need to compute eigenvalue decompositions with $m \times m$ matrices, where *m* is the dimension of the simulation, that is the number of inputs.

Active subspaces can be seen in the more general context of ridge approximation (see [25, 34]). In particular it can be proved that, under certain conditions, the active subspace is nearly stationary and it is a good starting point in optimal ridge approximation as shown in [11, 22].

## Numerical results

In this section we present the results of the complete pipeline, presented in the previous sections and in Fig. 1, applied to the DTMB 5415 hull.

The mesh is discretized with quadrilateral cells. The BEM uses bi-linear quadrilateral elements. This results in roughly 4000 degrees of freedom for each simulation realized. The high fidelity solver described in “Implementation of high fidelity potential solver based on the boundary element method” section is implemented in WaveBEM [29] using the deal.II library [2].

**Fig. 9:**
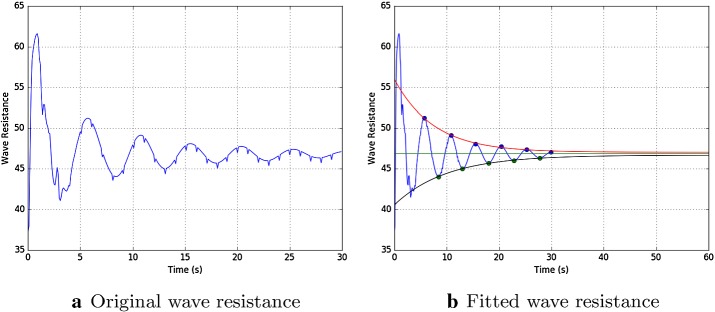
Plot **a** shows the original wave resistance simulated for 30 s. Then plot **b** depicts, after a filter has been applied, the exponential fitting of the maximums and minimums and the average at regime for 60 s

The solver, after reading the CAD file of the deformed geometry, simulates the behaviour of the hull for 30 s. To further speedup the computations, the total resistance computed as in Eq. (17) is extrapolated to obtain the total resistance at the final, steady state regime, with an error in the order of 0.1%. In Fig. 9 we can see the original simulation of the total resistance for the first 30 s and then the extrapolation we have done after a proper filtering of the data. We fit the maximums using the following function: $a e^{-b x} + c$. For the minimums we use $-a e^{-b x} + c$. Then we set the approximated wave resistance to the average of the two at infinity.

Let us recall that the parameter space is a $m = 8$ dimensional space. The parameters are showed in Table 1. We remark that the first six are geometrical parameters that produce the deformation of the original domain, while the last two are structural and physical parameters—the displacement and the velocity of the hull —. The PyGeM open source package is used to perform the free form deformation [35].

We create a dataset with 130 different couples of input/output data. We split the dataset in two, creating a train dataset with 80% of the data, and a test dataset with the remaining 20%. That means that $N_{\text {train}}^{\text {AS}} = 104$ in Eq. (19). Even though it may be challenging to explore a 8 dimensional space, as reported in [10], heuristics suggest that this choice of $N_{\text {train}}^{\text {AS}}$ is enough for the purposes of the active subspaces identification described in “Parameter space reduction by active subspaces” section.

In order to construct the uncentered covariance matrix $\varvec{\varSigma }$, defined in Eq. (18), we use a Monte Carlo method as shown in Eq. (19), employing the software in [9]. Since we have only pairs of input/output data we need to approximate the gradients of the total wave resistance with respect to the parameters, that is $\nabla _{\varvec{\mu }} f$. We use a local linear model that approximates the gradients with the best linear approximation using 14 nearest neighbors. After constructing the matrix we calculate its real eigenvalue decomposition. Recalling “Parameter space reduction by active subspaces” section, since $m = 8$, we have that $\varvec{\varSigma } \in {\mathbb {M}} (8, {\mathbb {R}})$.

**Fig. 10:**
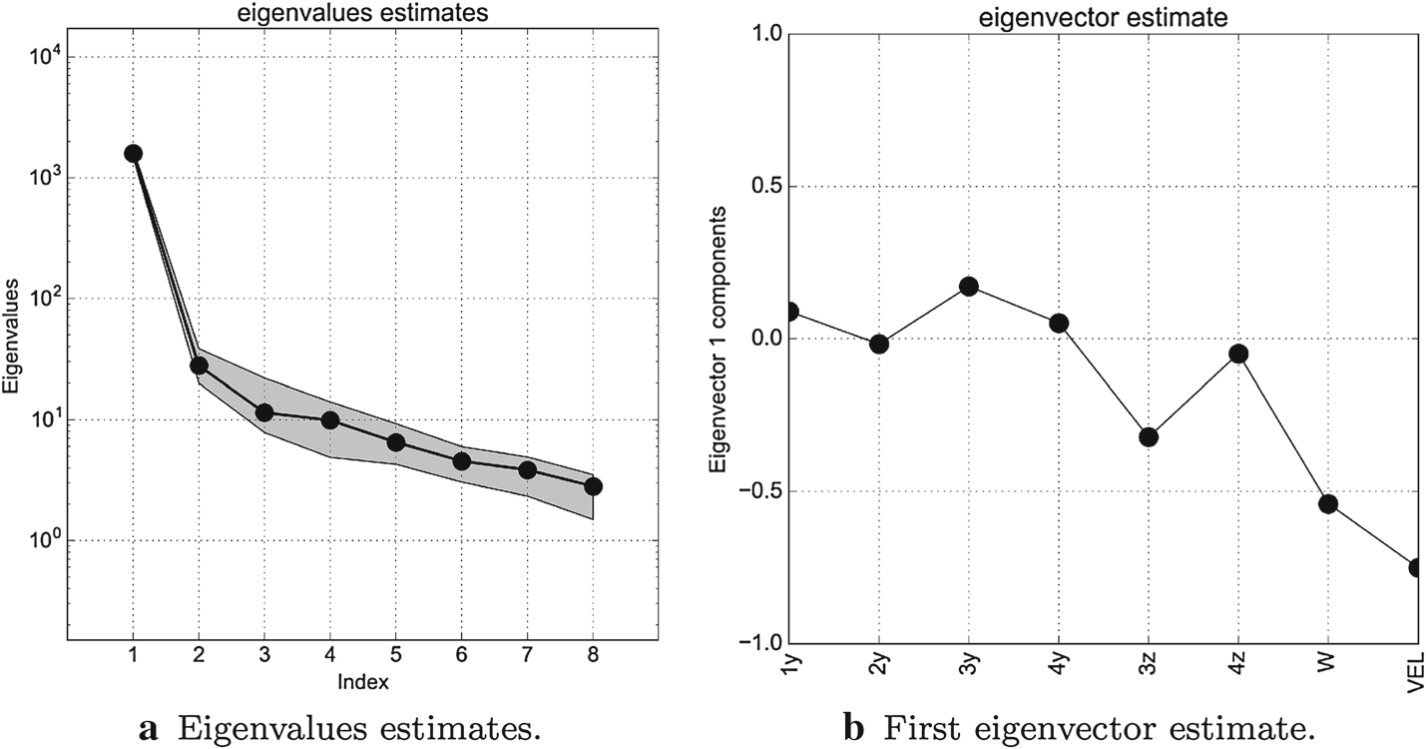
Plot **a** shows the eigenvalue estimates in block circles with the bootstrap intervals (grey region). The order-of-magnitude gaps between the eigenvalues suggest confidence in the dominance of the active subspace. Plot **b** shows the components of the eigenvector correspondent to the greatest eigenvalue

In Fig. 10a we see the eigenvalues of $\varvec{\varSigma }$ and the bootstrap intervals. Bootstrapping is the practice of estimating properties of a quantity (such as its variance) by measuring those properties when sampling from an approximating distribution. It relies on random sampling with replacement. The bootstrap intervals in grey are computed using 1000 bootstrap replicates randomly extracted from the original gradient samples. We underline the presence of a major gap between the first and the second eigenvalue and a minor one between the second and the third. This suggests the existence of a one dimensional subspace and possibly the presence of a two dimensional one. To better investigate the first case, in Fig. 10b we present the components of the eigenvector with index 1 that corresponds to the greatest eigenvalue, that is the matrix—in this case it is a vector—${\mathbf {W}}_1 \in {\mathbb {R}}^8$ of Eq. (21). Since they are the weights of the linear combination used to construct the active direction they provide useful informations. We can see that the major contributions are due to the velocity, the weight, and the depth of the hull. We underline that a weight that is almost zero means that the output function, on average, is almost flat along the direction identified by the corresponding parameter. This is a very useful information for a designer because in such a way he can deform the shape along prescribed directions without affecting the total wave resistance, allowing for example to store more goods inside the hull preserving the performances.

**Fig. 11:**
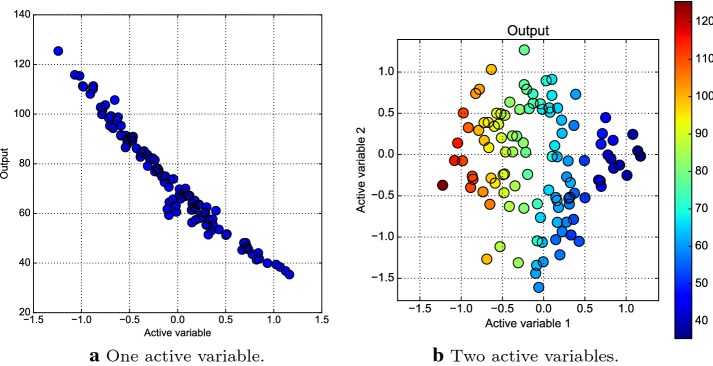
Sufficient summary plots for **a** one and **b** two active variables using the training dataset

In Fig. 11 we explore the training dataset, plotting the sufficient summary plot (see [13]) for one and two active variables. Sufficient summary plots are powerful visualization tools to identifying low-dimensional structure in a quantity that depends on several input variables. A scatter plot that contains all available regression information is called a sufficient summary plot. Recalling Eq. (22), Fig. 11 shows $f(\varvec{\mu })$ against $\varvec{\mu }_M = {\mathbf {W}}_1^T \varvec{\mu }$, where ${\mathbf {W}}_1$ contains the first one and two eigenvectors respectively. In particular each point represents the value of the output function for a particular choice of the parameters, mapped in the active subspace. The two plots confirm the presence of an active subspace of dimension one and two. The latter seems to capture the output function in a much finer way, but as we are going to show the gain in terms of error committed is not so big.

**Fig. 12:**
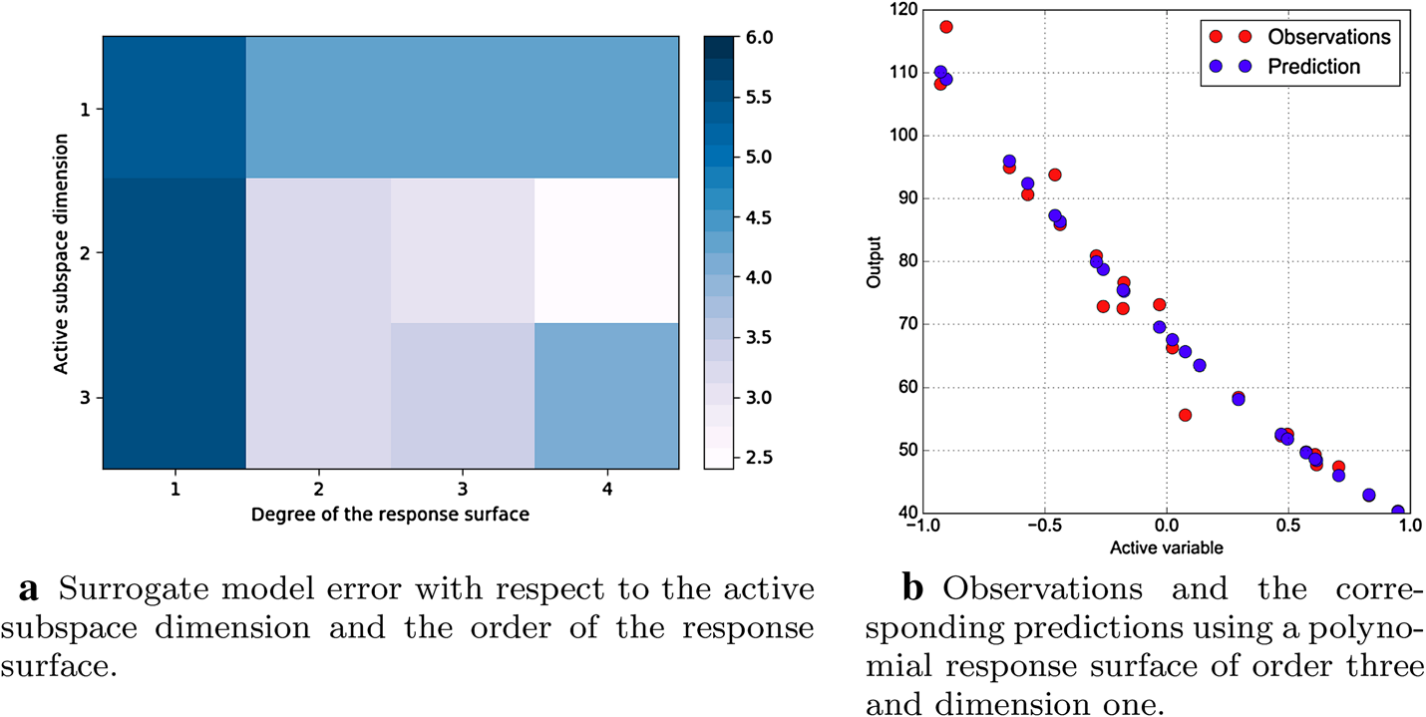
Plot **a** shows the relative error on the test dataset with respect to the active subspace dimension and the order of the response surface; plot **b** shows the observations of the test dataset and the corresponding predictions using a polynomial response surface of order three

We can compare the decay of the eigenvalues with the decay of surrogate model error on the test dataset shown in Fig. 12a. We project the data onto active subspaces of varying dimension, and construct a surrogate model with a least-squares-fit, global, multivariate polynomial approximation of different orders. Then we calculate the root-mean-square error of the test data against the surrogate. This error is scaled with respect to the range of the function evaluations, making it a relative error. We repeat this procedure 20 times constructing every time the uncentered covariance matrix of Eq. (19), since a Monte Carlo approximation is involved. Finally we take the average of the errors computed. Because we have a large amount of training data, we can expect the surrogate model constructed in a low dimension to be accurate if the data collapses into a manifold. Thus the test error is an indication of how well the active subspace has collapsed the data. In Fig. 12a are depicted the errors with respect to the active subspace dimension and the order of the response surface. The subspace dimension varies from 1 to 3, while the order of the response surface from 1 to 4. The minimum error is achieved using a two dimensional active variable and a response surface of degree 4 and it is $\approx 2.5\%$. Further investigations show that increasing the dimension of the active variable does not decrease significantly the error committed while the time to construct the corresponding response surface increases. This is confirmed by the marginal gains in the decay of the eigenvalues for active variables of dimension greater then three as shown in Fig. 10a. We can affirm that the active subspace of dimension one is sufficient to model the wave resistance of the DTMB 5415 if we can afford an error of approximately 4.5%. In particular in Fig. 12b we can see the predictions made with the surrogate model of dimension one and the actual observations. Otherwise, we can achieve a $\approx 2.5\%$ error if we take advantage of two active dimensions and a response surface of order four, preserving a fast evaluation of the surrogate model.

We want to stress the fact that the result is remarkable if we consider the heterogeneous nature of all the parameters involved. In the case of only geometrical parameters one can easily expect such a behaviour but considering also physical and structural ones make the result not straightforward at all. Moreover the evaluation of the response surface takes less than one s compared to the 12 h of a full simulation per single set of parameters on the same computing machine. This opens new potential approaches to optimization problems.

## Conclusions and perspectives

In this work we presented a numerical framework for the reduction of the parameter space and the construction of an optimized response surface to calculate the total wave resistance of the DTMB 5415 advancing in calm water. We integrate heterogeneous parameters in order to have insights on the more important parameters. The reduction both in terms of cost and time, remaining below the 4.2% of error on new unprocessed data, is very remarkable and promising as a new design interpreted tool. The methodological and computational pipeline is also easily extensible to different hulls and/or different parameters. This allows a fast preprocessing and a very good starting point to minimize the quantities of interest in the field of optimal shape design.

This work is directed in the development of reduced order methods (ROMs) and efficient parametric studies. Among others we would like to cite [7, 20, 37, 39] for a comprehensive overview on ROM and geometrical deformation. Future developments involve the application of the POD after the reduction of the parameter space through the active subspaces approach.
